# Construction of Aerobic/Anaerobic-Substrate-Induced Gene Expression Procedure for Exploration of Metagenomes From Subseafloor Sediments

**DOI:** 10.3389/fmicb.2021.726024

**Published:** 2022-01-13

**Authors:** Taisuke Wakamatsu, Saki Mizobuchi, Fumiaki Mori, Taiki Futagami, Takeshi Terada, Yuki Morono

**Affiliations:** ^1^Agricultural Sciences, Graduate School of Integrated Arts and Sciences, Kochi University, Kōchi, Japan; ^2^Geomicrobiology Group, Kochi Institute for Core Smaple Research, Japan Agency for Marine-Earth Science and Technology, Kōchi, Japan; ^3^Education and Research Center for Fermentation Studies, Faculty of Agriculture, Kagoshima University, Kagoshima, Japan; ^4^Marine Works Japan Ltd., Yokosuka, Japan

**Keywords:** substrate-induced gene expression, subseafloor, uncharacterized gene, anaerobic, halogenated compound, metagenome

## Abstract

Substrate-induced gene expression (SIGEX) is a high-throughput promoter-trap method. It is a function-based metagenomic screening tool that relies on transcriptional activation of a reporter gene *green fluorescence protein* (*gfp*) by a metagenomic DNA library upon induction with a substrate. However, its use is limited because of the relatively small size of metagenomic DNA libraries and incompatibility with screening metagenomes from anaerobic environments. In this study, these limitations of SIGEX were addressed by fine-tuning metagenome DNA library construction protocol and by using Evoglow, a green fluorescent protein that forms a chromophore even under anaerobic conditions. Two metagenomic libraries were constructed for subseafloor sediments offshore Shimokita Peninsula (Pacific Ocean) and offshore Joetsu (Japan Sea). The library construction protocol was improved by (a) eliminating short DNA fragments, (b) applying topoisomerase-based high-efficiency ligation, (c) optimizing insert DNA concentration, and (d) column-based DNA enrichment. This led to a successful construction of metagenome DNA libraries of approximately 6 Gbp for both samples. SIGEX screening using five aromatic compounds (benzoate, 3-chlorobenzoate, 3-hydroxybenzoate, phenol, and 2,4-dichlorophenol) under aerobic and anaerobic conditions revealed significant differences in the inducible clone ratios under these conditions. 3-Chlorobenzoate and 2,4-dichlorophenol led to a higher induction ratio than that for the other non-chlorinated aromatic compounds under both aerobic and anaerobic conditions. After the further screening of induced clones, a clone induced by 3-chlorobenzoate only under anaerobic conditions was isolated and characterized. The clone harbors a DNA insert that encodes putative open reading frames of unknown function. Previous aerobic SIGEX attempts succeeded in the isolation of gene fragments from anaerobes. This study demonstrated that some gene fragments require a strict *in vivo* reducing environment to function and may be potentially missed when screened by aerobic induction. The newly developed anaerobic SIGEX scheme will facilitate functional exploration of metagenomes from the anaerobic biosphere.

## Introduction

The remarkable advances of DNA sequencing technology (Mardis, 2017) have led to the accumulation of a vast amount of DNA sequence information, including that for uncultured microbes, i.e., metagenome-assembled genomes (Papudeshi et al., 2017) or single-cell genomes (Xu and Zhao, 2018). Currently, genomic DNA sequences of more than 300,000 microbes are registered in public databases, such as the National Center for Biotechnology Information (NCBI) database (as of June 2021)^1^. In parallel with the increasing amount of sequencing information, various pipelines for functional gene annotation, e.g., NCBI prokaryotic genome annotation pipeline (Tatusova et al., 2016) and BLAST programs (Altschul et al., 1990), have been developed. However, since sequence-based functional annotation usually relies on assessing the similarity with known sequences with experimentally confirmed function, the function of up to 40% of annotated genes in fully sequenced microbial genomes is unknown or, in some cases, with the function predicted but not confirmed (Baric et al., 2016). As an alternative approach to deciphering the function of unannotated DNA sequences, function-based metagenomics has been developed. This method is used to identify functional genes in shotgun metagenome libraries based on phenotypic changes of generated clones (for a review, see Ngara and Zhang, 2018). In the method, the clones are screened on agar plates for changes in colony morphology or the survival of host cells complemented with cloned genes is evaluated. Microplate screening is used when the conversion of substrate(s) to product(s) is detectable by absorbance or fluorescence intensity. As an advantage of these methods, the screening yields positive clones that contain functional genes. However, such phenotypic screens also result in a false-negative loss of positive clones that fail to function because of the formation of inclusion bodies or the absence of appropriate cofactors. Further, such screens are generally labor-intensive and time-consuming, because of a manual selection of positive clones with the desired enzymatic or other activity from the numerous clones from a metagenomic library (Berini et al., 2017).

Substrate-induced gene expression (SIGEX) system is another type of function-based metagenomic screen that uses a promoter trap-type vector and *Escherichia coli* as the host cell (Uchiyama et al., 2005). SIGEX assumes that general gene expression is induced by the substrates and/or metabolites of catabolic enzymes, and that regulatory elements (transcription factors or riboswitches) are often positioned in proximity to the catabolic genes. In the method, DNA fragments from environmental samples are inserted upstream of the gene for green fluorescence protein (*gfp*), and induction-positive clones are selectively isolated by fluorescence-activated cell sorting (FACS). The approach has several advantages: (a) positive clones harboring substrate-responsive genes are detected by the activation of a regulatory element, without relying on the functionality of the inserted genes; and (b) high-throughput isolation by FACS. To date, several groups have successfully applied SIGEX to screen metagenomes. For example, Uchiyama et al. (2005), Uchiyama and Watanabe (2008), Uchiyama and Miyazaki (2013), and Meier et al. (2016) analyzed groundwater and soil samples, respectively, and obtained genes induced by aromatic compounds. Futagami et al. (2013) and Wakamatsu et al. (2018) retrieved gene fragments from deep subseafloor microbes recovered from the Kumano forearc basin (Nankai Trough, Japan). The authors identified fragments responsive to organohalides, 2,4,6-tribromophenol (2,4,6-TBP) or 2,4,6-trichlorophenol (2,4,6-TCP), individually or by a mixture of 2,4,6-TBP, 2,4,6-TCP, and 2,4,6-triiodophenol, and metal ions (Ni^2+^, Ga^3+^, or Fe^3+^).

In the current study, we aimed to upgrade the capability of SIGEX analysis to include an expanded size of shotgun metagenomic library and to be applicable under anaerobic culture conditions. To expand the size of inserted DNA fragments in the shotgun metagenomic library, a topoisomerase (TOPO)-adapted vector was employed and the library construction protocol was fine-tuned. As a reporter gene, *evoglow* (Drepper et al., 2007) was introduced. The gene encodes a protein that acts as a fluorophore, emitting green fluorescence under both aerobic and anaerobic conditions. Two different shallow subseafloor sediment samples collected offshore Shimokita Peninsula and offshore Joetsu were used as environmental samples for this upgraded method. The differential expression patterns in the libraries upon aerobic and anaerobic induction with five aromatic compounds were examined. Then, one clone that responded to 3-chlorobenzoate induction only under anaerobic conditions was picked up as an example and its sequence showed no matching information to the Nucleotide Collection (nr/nt) database of NCBI. The established procedure will expand the knowledge of the function of environmental genomes otherwise unidentified by homology-based annotation procedures.

## Materials and Methods

### Optimization of Insert DNA Concentration for TOPO Ligation

The Ligation Calculator NEBioCalculator^2^ was used to calculate the insert DNA to vector molar ratio. The 16S rRNA gene fragments from *E. coli* JM109 Competent Cells (1.5 kbp long; Takara Bio Inc., Shiga, Japan) were amplified using TaKaRa Taq polymerase (Takara Bio Inc.) and universal primers 8F (5′-AGAGTTTGATCCTGGCTCAG-3′) and 1492R (5′-GGTTACCTTGTTACGACTT-3′) (Turner et al., 1999). For the experiment, 1 μL of the PCR products (0.54, 2.7, 5.4, 10, or 32 ng) purified using Wizard^®^ SV Gel and PCR Clean-Up System (Promega, Madison, WI, United States) was combined with the 1 μL of pCR™8/GW/TOPO (Invitrogen, Waltham, MA, United States, 2.8 kbp long; 10 ng, insert:vector molar ratios of 0.1:1, 0.5:1, 1:1, 2:1, or 6:1), 3 μL of sterile water, and 1 μL of four-fold diluted Salt Solution (Invitrogen), gently mixed, and left for 30 min at 25°C for the ligation reaction to take place. The ligated DNA was concentrated using DNA Clean & Concentrator-5 Kit (Zymo Research, Irvine, CA, United States) according to the manual protocol, to 6 μL in 10 mM Tris–HCl (pH 8.0). Then, 25 μL of *E. coli* MegaX DH10B T1 Electrocompetent Cells (Thermo Fisher Scientific, Waltham, MA, United States), the highest-efficiency electrocompetent cells that lack the *laqI^q^* gene, were transformed using 2 μL of the ligated DNA. Transformations were performed in MicroPulser Electroporator #1652100 (Bio-Rad, Hercules, CA, United States) at 1.8 kV, using a 1-mm-gap electroporation cuvette (Nepa Gene Co., Ltd., Chiba, Japan), followed by an immediate addition of 1 mL of Recovery Medium (Invitrogen). The samples were then incubated with shaking at 225 rpm for 1 h at 37°C. A small portion of cells was plated onto a Luria-Bertani (LB) agar plate containing 100 μg/mL spectinomycin to check the cloning efficiency.

### Optimization of DNA Enrichment After TOPO Ligation

For the experiment, 1 μL of 16S rRNA gene fragments from *E. coli* JM109 (5.4 ng), which were amplified using TaKaRa Taq^®^ polymerase and purified using Wizard SV Gel and PCR Clean-Up System, was combined with 1 μL of pCR8/GW/TOPO (10 ng, insert:vector molar ratio of 1:1), 3 μL of sterile water, and 1 μL of four-fold diluted Salt Solution, gently mixed, and left for 30 min at 25°C for the ligation reaction to take place. The DNA enrichment method was optimized by comparing ethanol precipitation and column purification methods. For DNA enrichment by ethanol precipitation, 50 μL of the ligated DNA in 10 mM Tris–HCl (pH 8.0), 5 μL of 3 M sodium acetate (pH 5.2), 1 μL of ethachinmate (NIPPON GENE Co., Ltd., Tokyo, Japan), and 125 μL of 99.9% ethanol were mixed, and the mixture was left for 1 h on ice. After centrifugation at 20,379 × *g* for 30 min at 4°C, the supernatant was removed and 300 μL of 70% ethanol was added. The solution was again centrifuged at 20,379 × *g* for 30 min at 4°C. The supernatant was removed, the precipitate was dried for 5 min, and then suspended in 6 μL of 10 mM Tris–HCl (pH 8.0). For DNA enrichment by column purification, 50 μL of the ligated DNA in 10 mM Tris–HCl (pH 8.0) were concentrated using DNA Clean & Concentrator-5 Kit to 6 μL in 10 mM Tris–HCl (pH 8.0). Then, 25 μL of *E. coli* MegaX DH10B T1 Electrocompetent Cells were transformed with 2 μL of the ligated DNA. Transformations were performed in MicroPulser Electroporator #1652100 at 1.8 kV using a 1-mm-gap electroporation cuvette, followed by an immediate addition of 1 mL of Recovery Medium. The samples were then incubated with shaking at 225 rpm for 1 h at 37°C. A small portion of the cells was plated onto an LB agar plate containing 100 μg/mL spectinomycin to check the cloning efficiency.

### Construction of Substrate-Induced Gene Expression Libraries

Marine sediment samples were collected in the course of two research cruises: cruise KY11-E06, offshore Shimokita Peninsula, Japan, with R/V Kaiyo, in November 2011 [dive#1339, 41°10′35.9″N, 142°12′1.97″E (Site C9001D), 1180 meters below sea level]; and cruise NT13-15, at the Joetsu Knoll, offshore Joetsu, Japan, with R/V Natsushima, in July 2013 (dive#1553, 37°31.234′N, 137°57.979′E, 1162 meters below sea level). Approximately 30-cm sediment cores were collected by 60-cm push corer. After excision into depth-specific slices, the sediment cores were frozen at –80°C until further processing in an onshore laboratory.

Bulk sediment DNA was extracted from 10 g of each frozen sediment from the depth of 0–5 cm below the seafloor for the KY11-E06 core (KY11-E06_0–5), and 0–10 cm below the seafloor for the NT13-15 core (NT13-15_0–10), by using PowerMax^®^ Soil DNA Isolation Kit (MO BIO Laboratories, Carlsbad, CA, United States) with slight modifications. Briefly, the PowerMax^®^ Soil PowerBead Solution (MO BIO Laboratories) was replaced by a 1:1 (w/w) mix of 0.1 mm and 0.5 mm zirconia beads (Yasui Kikai, Osaka, Japan), and the mixture was shaken by using Multi Beads Shocker (Yasui Kikai). After isopropanol precipitation, the precipitated DNA was washed with 70% ethanol and suspended in 50 μL of TE buffer (pH 8.0). NucleoMag^®^ NGS Clean-up and Size Select (Takara Bio Inc.) was then used to selectively recover DNA fragments longer than 800 bp, and the recovered fragments were suspended in 50 μL of 10 mM Tris–HCl (pH 8.0). The size selection process was repeated twice to ensure the removal of short fragments. Then, the fragments were end-polished using KOD DNA polymerase (Toyobo, Osaka, Japan) and 10 × Blunting Buffer (Toyobo), and purified using Wizard^®^ SV Gel and PCR Clean-Up System. The 5′- and 3′-termini of the DNA fragments were dephosphorylated using antarctic phosphatase (New England Biolabs, Ipswich, MA, United States) and purified using Wizard^®^ SV Gel and PCR Clean-Up System. The 3′-terminus of the fragments were dA-tailed using TaKaRa Taq polymerase. Finally, the DNA fragments were concentrated using DNA Clean & Concentrator-5 Kit in 6 μL of 10 mM Tris–HCl (pH 8.0).

**FIGURE 1:**
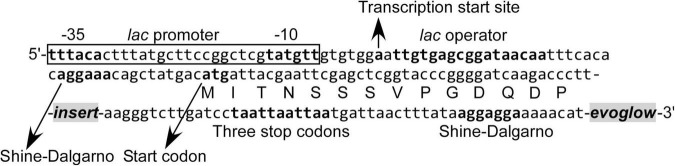
Nucleotide and amino acid sequence in the vicinity of DNA fragment inserted into the pK18evoglow-TOPO vector.

The modified promoter-trap vector pK18evoglow was constructed as described by Uchiyama et al. (2005) and Uchiyama and Watanabe (2008) with pK18 (Pridmore, 1987) as the starting vector and pGLOW-FBs2 (Jena Bioscience, Jena, Germany) as the source of *evoglow* (Drepper et al., 2007). TOPO adaptation of the pK18evoglow vector was done by Invitrogen. For library construction, 1 μL of DNA fragments prepared as described above (19 ng) was combined with 1 μL of pK18evoglow-TOPO vector (Invitrogen, Figure 1; 3.1 kbp long, 10 ng; assuming an insert length of 3.0 kbp, the insert:vector molar ratio was 2:1), 3 μL of sterile water, and 1 μL of four-fold diluted Salt Solution, gently mixed, and left for 30 min at 25°C for ligation reaction to proceed. The ligated DNA was concentrated using DNA Clean & Concentrator-5 Kit in 6 μL of 10 mM Tris–HCl (pH 8.0). Then, 25 μL of *E. coli* MegaX DH10B T1 Electrocompetent Cells were transformed using 2 μL of the ligated DNA. Transformations were performed using MicroPulser Electroporator #1652100 at 1.8 kV, in a 1-mm-gap electroporation cuvette, followed by an immediate addition of 1 mL of Recovery Medium and incubation at 37°C with shaking at 225 rpm for 1 h. Overall, five transformations were performed for each library, and a small portion of cells were plated onto an LB agar plate containing 50 μg/mL kanamycin to check the cloning efficiency. The SIGEX library was cultured at 37°C in 250 mL of LB medium supplemented with 50 μg/mL kanamycin to an optical density at 600 nm (OD_600_) of 0.6, and then the cells were stored at –80°C with 20% (v/v) glycerol.

The microbial community structure of extracted DNA was determined by amplifying approximately 300-bp fragments of the V4 variable region of the 16S rRNA gene by PCR with TaKaRa Ex Taq DNA polymerase (Takara Bio Inc.) and universal primers 515F (5′-TGYCAGCMGCCGCGGTAA-3′) and 806R (5′-GGACTACHVGGGTWTCTAAT-3′) (Caporaso et al., 2011). First-round PCR amplicons with 27 cycles were quantified and purified by gel electrophoresis, and then indexed and barcoded during the second round of PCR with 8 cycles. The amplicons were sequenced using the MiSeq platform (2 × 300 bp, Illumina, San Diego, CA, United States) and MiSeq Reagent Kit v3 (Illumina) at Bioengineering Lab. Co., Ltd. (Kanagawa, Japan). Raw MiSeq data were analyzed using Quantitative Insights into Microbial Ecology (Qiime2 v2020.11; Bolyen et al., 2019). For raw sequence data, the primer sequences were trimmed using the trim-paired function of the cutadapt Qiime2 plugin (Martin, 2011). Following primer sequence removal, the denoise-paired function of dada2 plugin (Callahan et al., 2016) was used for quality control, including chimera removal. Finally, the amplicon sequence variants were classified using the SILVA 138 SSU Ref NR 99 database (Quast et al., 2013), using RESCRIPt plugin (Bokulich et al., 2018; Robeson et al., 2020) trained using feature-classifier fit-classifier-naive-bayes function. At the visualization step, processing and quality control of the reads were performed using R (v4.0.4, R Core Team, 2020). The “phyloseq” (McMurdie and Holmes, 2013), “ggplot2” (Wickham, 2016), and “microViz”(Barnett, 2021) packages were used to create bar plots. Each of the extracted DNA contained Proteobacteria (KY11-E06_0–5: 25.6%, NT13-15_0–10: 31.3%) and Desulfobacterota (KY11-E06_0–5: 25.4%, NT13-15_0–10: 20.0%) as the most dominant phyla, followed by Bacteroidota (KY11-E06_0–5: 10.7%, NT13-15_0–10: 9.4%) in both DNA samples (Supplementary Figure 1). Gammaproteobacteria from the phyla Proteobacteria were the most dominant class (KY11-E06_0–5: 23.7%, NT13-15_0–10: 21.4%). Archaeal 16S rRNA was not detected to any great extent (KY11-E06_0–5: 2.4%, NT13-15_0–10: 5.5%) and no unassigned phylotypes were retrieved from the samples. A major difference between the two communities was that the KY11-E06_0–5 sample contained DTB120 (1.3%), while NT13-15_0–10 contained Latescibacterota (0.8%).

### Induction Under Aerobic Conditions

Clones in the SIGEX master library were grown with shaking at 130 rpm to OD_600_ of 0.1 at 37°C in LB medium supplemented with 50 μg/mL kanamycin. Then, 200 μL of the culture were transferred to 5 mL of dLB medium [pH 7.1, adjusted with NaOH; 0.25% (w/v) LB broth (Miller), 40 mM MOPS-NaOH, and 0.2% (w/v) maltose] supplemented with 20 μg/mL kanamycin and 1 mM substrate (benzoate, 3-chlorobenzoate, 3-hydroxybenzoate, phenol, 2,4-dichlorophenol, or IPTG). The culture was incubated with shaking at 130 rpm overnight to OD_600_ of 0.3 at 37°C. It was then diluted in phosphate-buffered saline (PBS; pH 7.2) to OD_600_ of 0.01. Histograms of the fluorescence of 1.00 × 10^6^ cells were obtained using Gallios flow cytometer with the 488-nm laser (Beckman Coulter, Brea, CA, United States). The data were analyzed using Kaluza Analysis Software (version 2.1, Beckman Coulter).

### Induction Under Anaerobic Conditions

Clones in the SIGEX master library were grown with fumarate respiration to OD_600_ of 0.1 at 37°C in N_2_-substituted MMYPF medium [pH 7.1, 0.8% (w/v) K_2_HPO_4_, 0.12% (w/v) KH_2_PO_4_, 0.75% (w/v) sodium pyruvate, 0.05% (w/v) trisodium citrate, 0.08% (w/v) disodium fumarate, 0.01% (w/v) MgSO_4_⋅7H_2_O, and 0.2% (w/v) yeast extract] containing 40 mM MOPS-NaOH, 0.0001% (w/v) resazurin sodium salt, 0.02% (w/v) sodium sulfide (pH 7.1), and 50 μg/mL kanamycin. Then, 200 μL of the culture were transferred to 5 mL of N_2_-substituted and 10-fold diluted MMYPF medium (pH 7.1) supplemented with 40 mM MOPS-NaOH, 0.0001% (w/v) resazurin sodium salt, 0.02% (w/v) sodium sulfide (pH 7.1), 20 μg/mL kanamycin, and 1 mM substrate (benzoate, 3-chlorobenzoate, 3-hydroxybenzoate, phenol, or 2,4-dichlorophenol). The culture was then incubated overnight to OD_600_ of 0.1 at 37°C. Resazurin in both the pre-culture and the main culture media was colorless. The culture was diluted in PBS (pH 7.2) to OD_600_ of 0.01, and histograms of the fluorescence of 1.00 × 10^6^ cells were obtained using Gallios flow cytometer with the 488-nm laser. The data were analyzed using Kaluza Analysis Software.

### Isolation of 3-Chlorobenzoate-Responsive Clones From the KY11-E06_0–5 Library Induced Under Anaerobic Conditions

Cells from the KY11-E06_0–5 library were grown at 37°C under anaerobic conditions to OD_600_ of 0.1. Then, 1 mM 3-chlorobenzoate was added as an inducer and the cells were incubated for an additional 11 h at 37°C. The culture was diluted in PBS (pH 7.2) to OD_600_ of 0.01. Fluorescence-positive clones were sorted as a single cell into 384-well plates (781162, Greiner Bio-One, Monroe, NC, United States) containing 80 μL of LB medium supplemented with 20 μg/mL kanamycin using MoFlo XDP cell sorter (Beckman Coulter). Substrate induction analysis of a clone of interest was performed as for the SIGEX libraries, except that the number of cells analyzed using the Gallios flow cytometer (Beckman Coulter) was 2.00 × 10^5^ cells.

### DNA Sequencing and Prediction of Gene Function

Sequence analysis of DNA insert in a clone identified as positive for induction by 3-chlorobenzoate under anaerobic conditions was done using the Sanger method at GENEWIZ Inc (Tokyo, Japan). The obtained nucleotide sequence data were analyzed using the NCBI programs BLASTN and BLASTX (Altschul et al., 1990), and ORFfinder (Wheeler et al., 2003). The potential promoter region was predicted by FGENESB (Solovyev and Salamov, 2011), and RNA secondary structure was predicted using Riboswitch Scanner (Mukherjee and Sengupta, 2016). StackDPPred program (Mishra et al., 2019), which utilizes features extracted from the position-specific scoring matrix and residue specific contact-energy, was used for the prediction of potential DNA-binding protein from an amino acid sequence.

## Results

### Optimization of the Protocol for Aerobic/Anaerobic-Compatible Substrate-Induced Gene Expression Library Construction

To expand the environmental metagenome coverage, we here optimized the steps of the protocol for SIGEX metagenome library construction. Three major steps were considered for the optimization, namely: (a) the design of plasmid vector with high DNA fragment insertion efficiency; (b) quality of the inserted DNA fragments; and (c) transformation efficiency of the host *E. coli* cells. Further, in the current study, an aerobic/anaerobic-compatible DNA vector with *evoglow* as a reporter gene was designed. The vector was also adapted by TOPO at its 3′ end of dT-overhang. A test of vector self-ligation, without DNA insert, resulted in a greatly reduced number of transformants [1.6 × 10^4^ colony-forming unit (cfu)/μg-vector]. The molar insert:vector ratio for TOPO-based ligation was also optimized. As shown in Supplementary Figure 2, 2:1 mixture of the DNA insert and vector resulted in the highest transformation efficiency.

The next optimization step concerned the DNA size selection, which is the most important step in plasmid library preparation. In previous reports, inserted DNA was size-selected by electrophoresis and gel excision (Futagami et al., 2013; Wakamatsu et al., 2018). Although a TOPO-vector with a dT-overhang at its 3′ end was used in the above-mentioned studies, clones with green fluorescence, which indicated that no DNA insert was introduced into the plasmid, were frequently observed. Since the vector did not yield many self-ligation transformants, the reason for the high frequency of green clones was speculated to be due to the introduction of short DNA fragments into the vector. In the original SIGEX scheme described by Uchiyama et al. (2005) and Uchiyama and Watanabe (2008), green fluorescent clones are eliminated at the first stage of sorting. However, that step requires an intensive sorting effort (at least three times of the variation of library clones), and we therefore aimed to eliminate short DNA fragments (potential inserts) from the prepared pool of inserts. To do this, a magnetic bead-based NucleoMag^®^ NGS Clean-up and Size Select kit was used to recover long DNA fragments. This kit has been originally designed to recover DNA molecules of a certain size-range (typically, 150–800 bp) for library preparation for next-generation sequencing. However, the beads are unique in that the size of the binding DNA varies depending on the ratio of the DNA solution and beads. We therefore used the kit to selectively recover large DNA fragments (over 800 bp). Two rounds of selection allowed for a successful recovery of long DNA fragments (Supplementary Figure 3). Elimination of shorter DNA fragments from the prepared pool of inserts was further confirmed by 100% insertion efficiency into the plasmid vector (56 clones tested).

We next optimized the electroporation transformation procedure. In the previous studies (Futagami et al., 2013; Wakamatsu et al., 2018), ethanol precipitation was used to concentrate the ligation products. However, there were problems of arching or high current-induced reduction of transformation efficiency happened upon the electroporation step. By changing the procedure for ligation product concentration from ethanol precipitation to column-based concentration, the transformation efficiency was increased approximately 10 times, from 1.1 × 10^7^ to 1.2 × 10^8^ cfu/μg-vector.

By applying the above-mentioned optimizations, shotgun SIGEX libraries with an average insert fragment length of 2 kbp and approximately 3 × 10^6^ clones in variation were successfully constructed. Two sets of DNA inserts extracted from subseafloor sediments (KY11-E06_0–5, from offshore Shimokita, Pacific Ocean, and NT13-15_0–5, from offshore Joetsu, Japan Sea) were used. The insert length (0.8–8 kbp) was confirmed by restriction enzyme digestion (*Eco*RI/*Hin*dIII) of plasmids from randomly selected 56 clones (28 clones from each library). The sum length of DNA inserts (approximately 6 Gbp for both libraries) exceeded that of our previous attempts (0.05 Gbp) (Futagami et al., 2013; Wakamatsu et al., 2018), and those reported by Uchiyama et al. (2005), Uchiyama and Watanabe (2008), Uchiyama and Miyazaki (2013), (0.1 Gbp) and by Meier et al. (2016) (1 Gbp).

### Substrate Induction Experiments Using the Substrate-Induced Gene Expression Libraries

**FIGURE 2:**
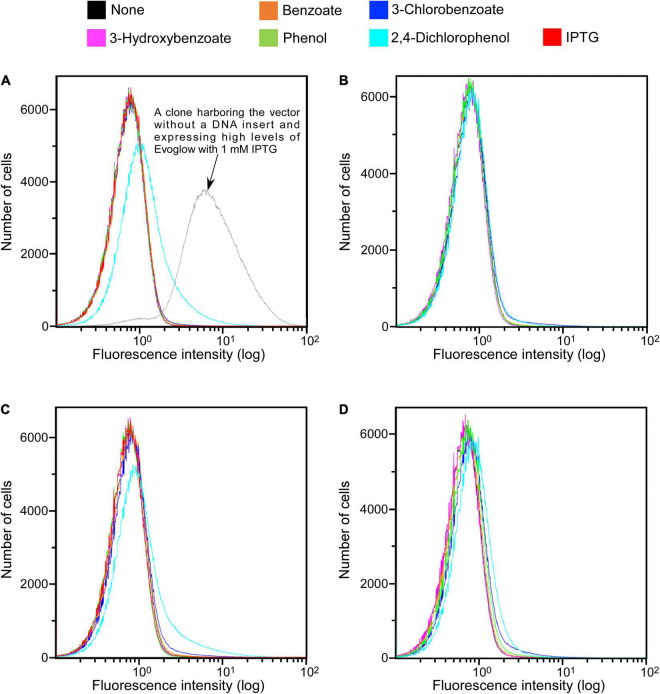
SIGEX library expression histograms of Evoglow. Histograms of fluorescence of 1.00 × 10^6^ cells were obtained using Gallios flow cytometer after cell exposure to the indicated compounds. The fluorescence intensity (log) was displayed up to 1.00 × 10^2^. **(A)** KY11-E06_0–5 library (aerobic conditions); **(B)** KY11-E06_0–5 library (anaerobic conditions); **(C)** NT13-15_0–10 library (aerobic conditions); **(D)** NT13-15_0–10 library (anaerobic conditions).

The functionality of Evoglow as a reporter protein was next tested in aerobic and anaerobic cultures. A clone harboring the vector without a DNA insert showed a clearly distinguishable green fluorescence (Figure 2A). Its fluorescence intensity did not change under aerobic and anaerobic conditions.

The constructed libraries were subsequently used in substrate induction experiments for SIGEX screening, in which induction-positive clones co-expressed Evoglow protein and showed green fluorescence. Flow cytometry analysis of negative control, with *E. coli* 16S rRNA gene inserted into the vector, showed a faint green fluorescence (only distinguishable by flow cytometry) under both aerobic and anaerobic conditions. This faint fluorescence was set as baseline expression, and any expression brighter than the baseline was considered as a positive (induced) expression. To examine the library quality, the two libraries were induced using various substrates, and the fluorescence signal was compared to those of the positive and negative controls (Figure 2 and Table 1). The addition of IPTG under aerobic conditions, the inducer of *lac* operon (Figure 1), did not induce Evoglow expression, confirming the absence of self-ligation or small inserts in the libraries. Further, green fluorescent clones were not detected in the absence of the inducing substrates.

**TABLE 1:** Percentage of clones with green fluorescence in response to specific substrates, calculated for an area with 1.00 × 10^3^ cells that emitted high fluorescence when no substrate was added per 1.00 × 10^6^ cells detected by flow cytometry.

KY11-E06_0–5 library		
**Substrate**	**Aerobic conditions clones (%)**	**Anaerobic conditions clones (%)**

Benzoate	0.22 ± 0.02	0.11 ± 0.04
3-Chlorobenzoate	0.49 ± 0.09	1.12 ± 0.12
3-Hydroxybenzoate	Not detected	0.02 ± 0.00
Phenol	Not detected	0.02 ± 0.00
2,4-Dichlorophenol	10.79 ± 0.48	0.56 ± 0.05
IPTG	Not detected	Not determined

**NT13-15_0–10 library**		

**Substrate**	**Aerobic conditions clones (%)**	**Anaerobic conditions clones (%)**

Benzoate	0.12 ± 0.03	0.10 ± 0.03
3-Chlorobenzoate	0.71 ± 0.09	1.10 ± 0.12
3-Hydroxybenzoate	Not detected	0.02 ± 0.00
Phenol	Not detected	0.16 ± 0.05
2,4-Dichlorophenol	4.70 ± 0.41	0.58 ± 0.09
IPTG	Not detected	Not determined

*Values and errors represent the average and standard deviation of three independent experiments, respectively. The number of clonal variations was approximately 3 × 10^6^ for both libraries. Not detected indicates 0.01% or less.*

A comparison of library fluorescence under aerobic and anaerobic conditions revealed different induction responses in the two SIGEX libraries (Table 1). The libraries showed a more pronounced induction response to benzoate (0.12–0.22%) and 2,4-dichlorophenol (4.70–10.79%) under aerobic conditions than under anaerobic conditions, while a more pronounced induction response was apparent in the presence of 3-chlorobenzoate (1.10–1.12%), 3-hydroxybenzoate (0.02%), and phenol (0.02–0.16%) under anaerobic conditions than under aerobic conditions. For 3-hydroxybenzoate and phenol, a slight induction was observed only under anaerobic conditions, implying that the devised anaerobic SIGEX scheme could be used to retrieve clones that harbor gene products that are not functional under and do not respond to aerobic conditions. The response to 3-chlorobenzoate was more pronounced than those to benzoate and 3-hydroxybenzoate, and the response to 2,4-dichlorophenol was more pronounced than that to phenol under both aerobic and anaerobic conditions in both libraries. This clearly demonstrates that subseafloor microbial genes analyzed in the current study were more responsive to chlorinated compounds than to non-chlorinated compounds. More than eight-fold higher responses to 2,4-dichlorophenol were observed under aerobic conditions (4.70–10.79%) than under anaerobic conditions (0.56–0.58%).

### Analysis of an Isolated Clone Responsive to 3-Chlorobenzoate Under Anaerobic Conditions

**FIGURE 3:**
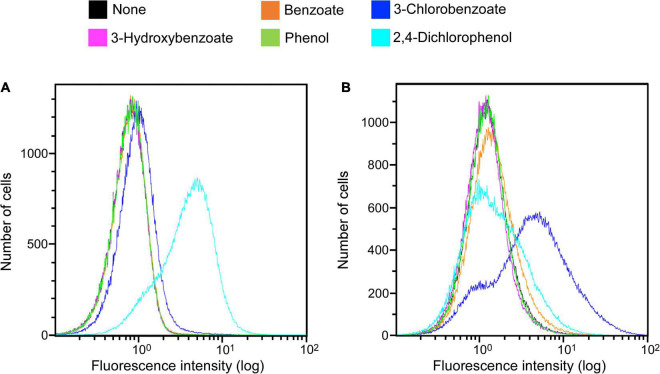
Clone expression histograms of Evoglow. The clone was isolated from the KY11-E06_0–5 library after induction with 3-chlorobenzoate under anaerobic conditions. Histograms of fluorescence of 2.00 × 10^5^ cells were obtained using Gallios flow cytometer after cell exposure to the indicated compounds. The fluorescence intensity (log) was displayed up to 1.00 × 10^2^. **(A)** Aerobic conditions; **(B)** anaerobic conditions.

Clones induced by 3-chlorobenzoate from the KY06-E06_0–5 library under anaerobic conditions were sorted. One of the isolated clones was picked up for the detailed examination of its response specificity to aromatic compounds. Among the five aromatic substrates tested (benzoate, 3-chlorobenzoate, 3-hydroxybenzoate, phenol, and 2,4-dichlorophenol), the response to 3-chlorobenzoate under anaerobic condition was significant, while the response was much weaker under aerobic conditions (Figure 3). On the other hand, the clone showed a more pronounced induction of fluorescence in response to 2,4-dichlorophenol upon aerobic incubation than during anaerobic incubation. Although the final OD_600_ of approximately 0.3 at the end of aerobic induction was typically observed, the final OD_600_ reached 0.1 after induction by 2,4-dichlorophenol, indicating cellular toxicity of the expressed protein and/or the metabolite of 2,4-dichlorophenol. For the other three non-chlorinated aromatic compounds, the clone did not show a clear induction response, while a slight induction was observed in the presence of benzoate under anaerobic conditions.

**FIGURE 4:**
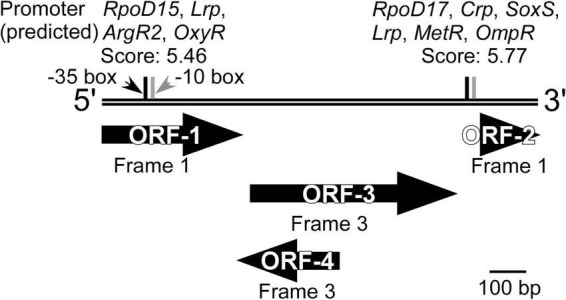
Overview of the DNA inserted into the clone isolated from the KY11-E06_0–5 library after induction with 3-chlorobenzoate under anaerobic conditions. ORFs identified by ORFfinder and BLASTX, and potential promoters predicted using FGENESB and with threshold values >3.00 are shown.

Sequencing of the DNA insert in the selected clone, followed by BLASTN search, revealed that the inserted DNA fragment (1,114 bp) did not share similarity with any sequence deposited in the Nucleotide Collection (nr/nt) database of NCBI, when analyzed as a full-length sequence. BLASTX and ORFfinder analyses were then used to identify open reading frames (ORFs) and determine any amino acid-based sequence similarity. Four ORFs that encode an amino acid stretch of more than 50 residues (ORF-1 to ORF-4, Figure 4) were identified, two of which had homologies to the sequences in the database. It should be noted that ORF1 (127 a.a.) and ORF2 (52 a.a.) were most likely not complete ORFs in the original genome because they contained the start and stop codons of the vector, respectively (Figure 1). ORF1 and ORF3 (189 a.a.) showed homology to a multidrug transporter (top hit: a protein from the aerobic *Cyclobacteriaceae* bacterium, 212–328 a.a., 72% identity, *E*-value; 6e^–55^) and carbonic anhydrase (top hit: a protein from the anaerobic *Ignavibacteriae* bacterium, 1–187 a.a., 75% identity, *E*-value; 5e^–87^), respectively. ORF2 and ORF4 (94 a.a.) were unknown proteins, but the StackDPPred program identified those ORFs as having DNA-binding potential. This suggested that one or both of these ORFs might act as transcription factors. A promoter search using the FGENESB identified potential promoter regions for the global regulators Crp (Salgado et al., 2013), Lrp (Unoarumhi et al., 2016), and OmpR (Shimada et al., 2015) immediately upstream of ORF2. The Riboswitch scanner did not detect any known riboswitch sequences.

## Discussion

This study presents the development of an improved SIGEX approach for anaerobic screening of genes from microbes dwelling in extreme habitats. The method improves the library coverage and allows the detection of novel genes with functions specific to the screened environments.

Various anaerobes thrive in diverse environments, such as the gastrointestinal tract of human and many animals (Hillman et al., 2017), extremely hot or salty habitats (Lowe et al., 1993), groundwater (Aburto et al., 2009), the soil (Ueki et al., 2018), and subseafloor sediments (Imachi et al., 2019). The subseafloor biosphere is one of the most challenging and understudied biospheres of microbial life on Earth despite its spatial distribution and large biomass (Whitman et al., 1998; Kallmeyer et al., 2012; LaRowe and Amend, 2015; Hoshino et al., 2020). A good example is the study by Gaboyer et al. (2015) who analyzed the metagenomes of subseafloor sediment samples collected from Canterbury Basin. About 35% of the metagenomic sequences did not match any known ORFs. It is also known that >50% of genes from microbial dark matter are neither functionally nor taxonomically annotated (Bernard et al., 2018). These indicate that additional approaches, such as the anaerobic SIGEX method developed herein, can be used to supplement the lack of knowledge on gene function and ecology of microbial life in extreme environments.

In parallel with the rapid development of DNA sequencing technology, function-based metagenomic approaches should be accordingly upgraded. One issue to be addressed is the coverage of environmental genomes. The advancement of sequencing capacity has allowed access to the low abundance (1%) microbes and part of the rare (0.1%) microbial biosphere (Pascoal et al., 2021). The shotgun metagenome library should also be expanded to cover those genes and perform a wide screening for complementing unknown functional information. The other issue to be addressed is the expression environment. All previous studies using SIGEX rely on *gfp*, which critically requires oxygen for fluorophore formation (i.e., to exhibit fluorescence; Reid and Flynn, 1997), as the reporter gene. Therefore, to date, induction analyses have been performed only under aerobic conditions. Although approximately a quarter of inducible genes identified by aerobic SIGEX screening shared similarity with anaerobic microbial genes (Futagami et al., 2013; Wakamatsu et al., 2018), transcription factors that do not function under oxidative conditions have been reported (Unden and Schirawski, 1997; Pop et al., 2004; Mesa et al., 2009). The potential of using *gfp* under anaerobic conditions was shown for a facultative anaerobic bacterium *Enterobacter aerogenes* under anaerobic culture conditions, by transferring the culture to aerobic conditions (Zhang et al., 2005). However, Wakamatsu et al. (2018) reported a failure of GFP fluorescence recovery using a facultative anaerobic bacterium *E. coli* as the host. These observations indicate the unsuitability of using *gfp* as a reporter gene for substrate induction under highly reducing anaerobic conditions. To expand the ability of SIGEX to explore unknown microbial functions, a reporter gene that is active under anaerobic conditions has been needed. In the current study, we constructed SIGEX libraries using a TOPO vector harboring *evoglow*, for efficient, high-coverage, and high-throughput functional screening of metagenomes from two subseafloor sediment samples. Evoglow is the protein that was genetically engineered from blue-light photoreceptors from *Bacillus subtilis* and *Pseudomonas putida* (Drepper et al., 2007). The *evoglow* was purposely engineered to create the fluorescent protein for anaerobic culture and its chromophore formation happens both under aerobic and anaerobic conditions. TOPO cloning allows for rapid DNA ligation and with higher insertion efficiency [reaction time of approximately 5 min and >95% ligation efficiency (Shuman, 1994) and information on the Thermo Fischer Scientific webpage] than those of conventional cloning using DNA ligase (1 h and approximately 80%, respectively). Further, we used TA cloning for efficient universal cloning that does not require restriction enzymes and for a reduced incidence of the self-ligation of the vector (Geng et al., 2006). By applying these optimizations, we constructed aerobic/anaerobic-compatible shotgun metagenomic SIGEX libraries of approximately 3 × 10^6^ clonal variations, with an average DNA insert size of 2 kbp, for a total of 6 Gbp of environmental metagenome inserts.

Of note, besides the library size, TOPO cloning greatly reduced the time required for the SIGEX library construction. Uchiyama et al. (2005) and Uchiyama and Watanabe (2008) used DNA ligase to insert metagenomic DNA into the plasmid vector. By placing *lac* promoter upstream of the DNA insert, clones with self-ligated empty vector or very short DNA inserts can be identified and removed by FACS. Clones without green fluorescence (i.e., clones with inserts) are then selectively sorted. However, this process has the inherent risk of losing the original variation of the metagenomic library. First, this type of sorting is based on random selection or pick up. Since there is a chance to sort the same clone more than twice, the sorting events required to keep the variation are not equal to the number of original clonal variations. Uchiyama and Watanabe (2008) sorted approximately five times as many cells as the number of clonal variations. Second, some clones lose viability during FACS. Indeed, Lopez and Hulspas (2020) reported “sorter-induced cell stress”. Although this term mainly refers to the mammalian cells, we also observed a reduction of live cell numbers after sorting, with only approximately half of the sorted *E. coli* cells surviving the process (Supplementary Table 1). To maintain the quality of SIGEX library, which critically affects SIGEX performance, a high number of sorting events are needed. Even with a cutting-edge cell sorter, the sorting of 10^6^–10^7^ cells requires a major investment of time and can be labor-intensive. Meier et al. (2016) used vector DNA without *lac* promoter and skipped the first FACS step; however, the authors nonetheless needed to exclude constitutively expressing clones after the second FACS step, during which substrate induction is performed. In the previous studies (Futagami et al., 2013; Wakamatsu et al., 2018), there was a major problem of a high percentage of clones constitutively expressing GFP, as high as 60–70% of the entire initial library. The use of TOPO-cloning, together with the effective removal of short DNA fragments resolved this problem. Further, a constitutive expression of *evoglow* was not detected, and the FACS step was not needed for SIGEX library construction. Together with the analysis of 56 randomly selected clones (28 clones from each library), which harbored long DNA inserts, the absence of Evoglow-expressing clones in the two library cultures induced by IPTG in the absence of substrate further confirmed the high quality of the constructed SIGEX libraries. Upon the addition of the inducing substrate, we detected induction-positive clones (Figure 2 and Table 1) and successfully isolated the clones by FACS. Although the quantum yield and extinction coefficient of Evoglow [0.39 and 12,500 M^–1^ cm^–1^ at 450 nm, respectively (Drepper et al., 2007)] are lower than those of enhanced GFP [0.60 and 55,000 M^–1^ cm^–1^ at 489 nm, respectively (Patterson et al., 2001)], the flow cytometric analyses revealed distinguishable fluorescence upon substrate induction of SIGEX libraries (Figure 2 and Table 1), and demonstrated the utility of the new SIGEX approach to expand the knowledge of anaerobic gene function.

The substrate induction analysis of the two SIGEX libraries by chlorinated aromatic compounds (Figure 2 and Table 1) also indicated that subseafloor microbes have potential to interact with halogenated aromatic compounds. Since halogenated compounds are generally recalcitrant and/or not metabolizable by aerobic microbes in the seawater column (King, 1988), they tend to be buried in marine subsurface sediments (Müller et al., 1996; Leri et al., 2010, 2015). In addition, significant reductive dehalogenation activities were observed in subseafloor sediments (Futagami et al., 2009, 2013; Atashgahi et al., 2018). Therefore, organohalide respiration is considered to be an important energy-yielding pathway in the subseafloor microbial ecosystem. Although benzoate and 2,4-dichlorophenol showed higher percentage of induction in aerobic condition, the other three compounds (3-chlorobenzoate, 3-hydroxybenzoate, and phenol) showed higher induction response in anaerobic condition (Table 1). To the best of our knowledge, there has been no report showing an inducing factor that requires a strictly *in vivo* reducing environment. Futagami et al. (2013) and Wakamatsu et al. (2018) have retrieved DNA fragments potentially originating from anaerobes, suggesting that the inducing factor can be stabilized in the intracellular environment of *E. coli* host cell (Pop et al., 2004; Gabor et al., 2006, 2008). However, the three compounds’ inductions observed under anaerobic conditions strongly indicated the presence of genes that only functions under strictly reducing conditions. The isolation of clone that responds to 3-chlorobenzoate only under anaerobic conditions further supports this notion. Substrate specificity analysis of the 3-chlorobenzoate-responded clone revealed that it also responds to 2,4-dichlorophenol upon aerobic induction but with no (little) response to the non-chlorinated compounds (benzoate, 3-hydroxybenzoate, and phenol) under aerobic and anaerobic conditions (Figure 3). Although it is not entirely clear whether the responses are due to the induced compound in its original chemical state, the result indicates that a region of the retrieved DNA fragment is responsible for a highly specific response to chlorinated compounds. Considering the substrate specificity of the induction response to 3-chlorobenzoate and 2,4-dichlorophenol (Figure 3), ORF2 and ORF4 (Figure 4) might act as transcription factors with different substrate-specificities. Further, the immediately upstream of ORF2, we identified several sequences recognized by transcriptional regulators; e.g., Crp, whose binding motif is similar to that of CprK, a transcriptional activator of organohalide respiration (Pop et al., 2004; Gabor et al., 2006, 2008). However, the other regions may be involved in the response to halogenated compounds. Further fragmentation analysis of the cloned DNA like the study by Uchiyama and Miyazaki (2013) will help to identify the regions that are essential for the response. Also, future combination with metagenomic analysis to obtain the relevant down- and upstream sequence information (Meier et al., 2016) and isolating many additional positive clones will further facilitate unveiling the unknown gene functions buried in natural environments.

## Conclusion

In the current study, we successfully developed and optimized a simple procedure for constructing large SIGEX libraries avoiding a massive FACS step by using metagenomic DNA extracted from subseafloor sediments. The use of *evoglow* reporter gene enabled the search of inducible DNA fragments even under anaerobic conditions. The SIGEX induction experiment under aerobic and anaerobic conditions involving five aromatic compounds revealed clear differences in the ratio of inducible clones under the conditions tested. The improved SIGEX method is a new approach for the prediction of microbial gene functions under reducing conditions, such as in subseafloor environments. In addition, the method is applicable to the exploration of novel biosensors that might have been missed in previous SIGEX studies relying on the aerobic *gfp* gene. Indeed, as a proof-of-concept, we retrieved a 3-chlorobenzoate-responsive clone. Although its induction mechanism specifically under anaerobic conditions is currently unclear, in-depth sequencing and accumulation of additional DNA fragments will help to elucidate it in the future.

## Data Availability Statement

The 16S rRNA gene amplicon sequence data generated in this study can be found in the DDBJ/ENA/GenBank Database under BioSample IDs SAMD00291243–SAMD00291244. All data were registered under BioProject ID PRJDB11436. The nucleotide sequence (1182 bp) from the start codon downstream of the *lac* promoter to the insert sequence, and three stop codons was deposited in the DDBJ/EMBL/GenBank database under accession number LC621909.

## Author Contributions

TW and YM conceived and designed the experiments and wrote the manuscript. TW, SM, FM, TT, and YM carried out the experiments and performed the analysis. TF revised the manuscript. All authors discussed the results and commented on the manuscript.

## Conflict of Interest

TT was employed by Marine Works Japan Ltd. The remaining authors declare that the research was conducted in the absence of any commercial or financial relationships that could be construed as a potential conflict of interest.

## Publisher’s Note

All claims expressed in this article are solely those of the authors and do not necessarily represent those of their affiliated organizations, or those of the publisher, the editors and the reviewers. Any product that may be evaluated in this article, or claim that may be made by its manufacturer, is not guaranteed or endorsed by the publisher.

## Footnotes

1
https://www.ncbi.nlm.nih.gov/genome/browse#!/overview/

2
https://nebiocalculator.neb.com/#!/ligation
