# Supplementary material for: Construction of Aerobic/Anaerobic-Substrate-Induced Gene Expression Procedure for Exploration of Metagenomes From Subseafloor Sediments
Source: Front Microbiol. 2022 Jan 13;12:726024. doi: 10.3389/fmicb.2021.726024 (PMC8793675; doi:10.3389/fmicb.2021.726024)
Supplement: Supplementary file 1 [file Data_Sheet_1.pdf]

Supplementary Material for

Construction of Aerobic/Anaerobic-Substrate-Induced Gene Expression Procedure for Exploration of Metagenomes From Subseafloor Sediments

Taisuke Wakamatsu[1], Saki Mizobuchi[1], Fumiaki Mori[2], Taiki Futagami[3], Takeshi Terada[4], Yuki Morono[2*]

[1]Agricultural Science, Graduate School of Integrated Arts and Sciences, Kochi University, Kōchi, Japan
[2]Geomicrobiology Group, Kochi Institute for Core Sample Research, Japan Agency for Marine-Earth Science and Technology (JAMSTEC), Kōchi, Japan
[3]Education and Research Center for Fermentation Studies, Faculty of Agriculture, Kagoshima University, Kagoshima, Japan
[4]Marine Works Japan Ltd., Kanagawa, Japan

***Correspondence:**
Yuki Morono
morono@jamstec.go.jp

1. Supplementary Figures and Tables

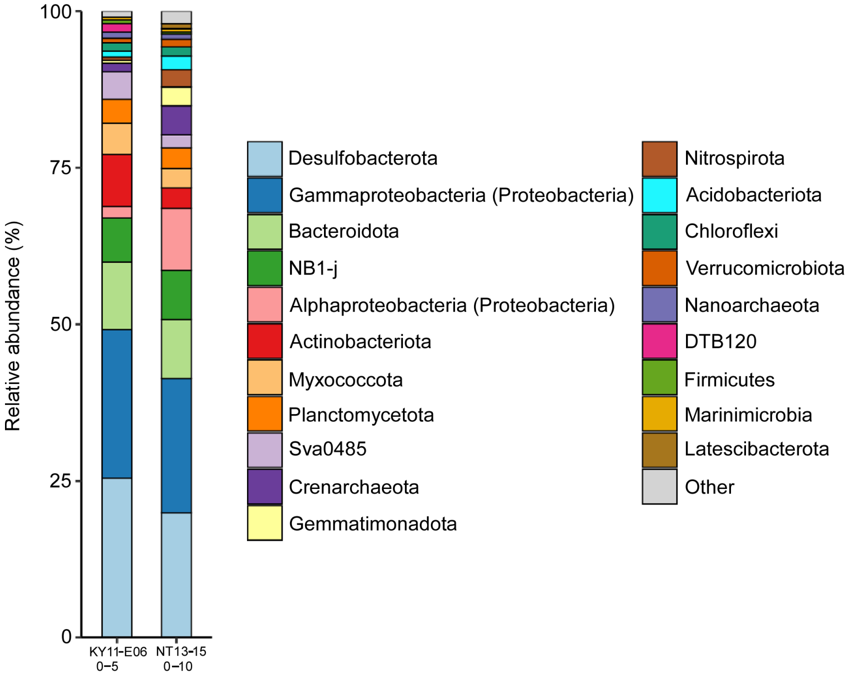

FIGURE S1. Microbial community structure of extracted DNA samples. Colors indicate different microbial phyla or classes.

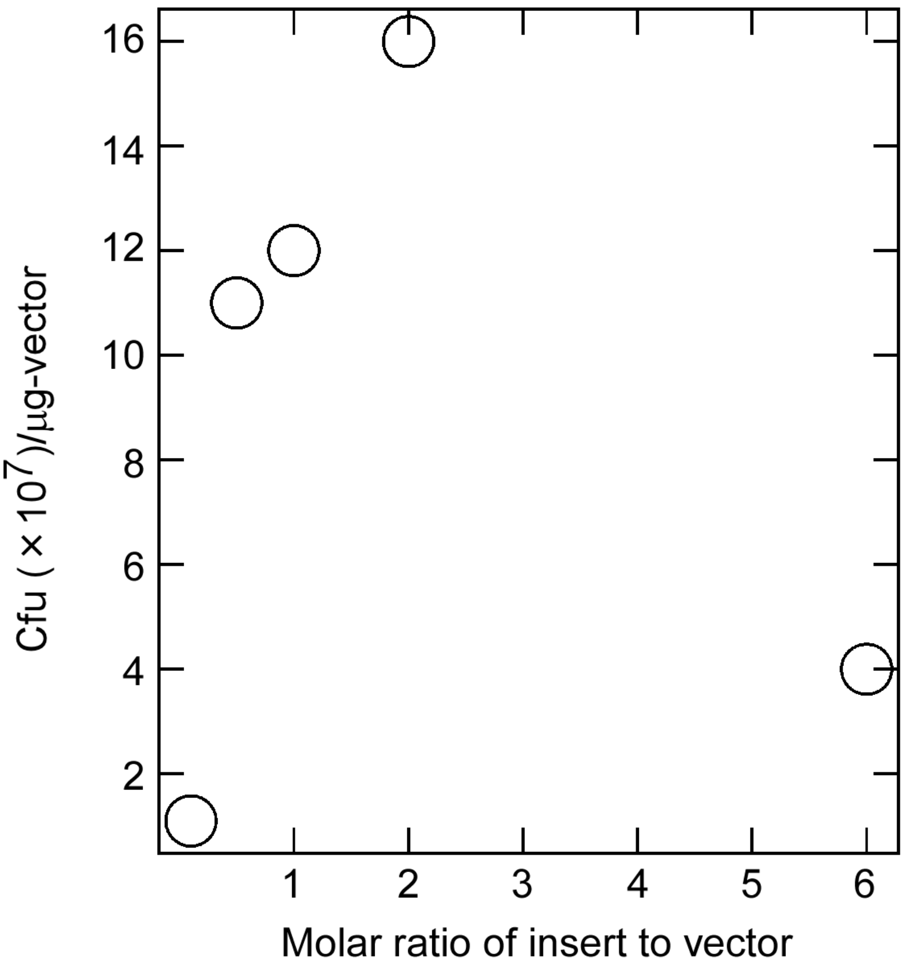

Figure S2. Effect of insert-to-vector molar ratio on ligation efficiency.
The vector DNA quantity was maintained at 10 ng in TOPO ligation.

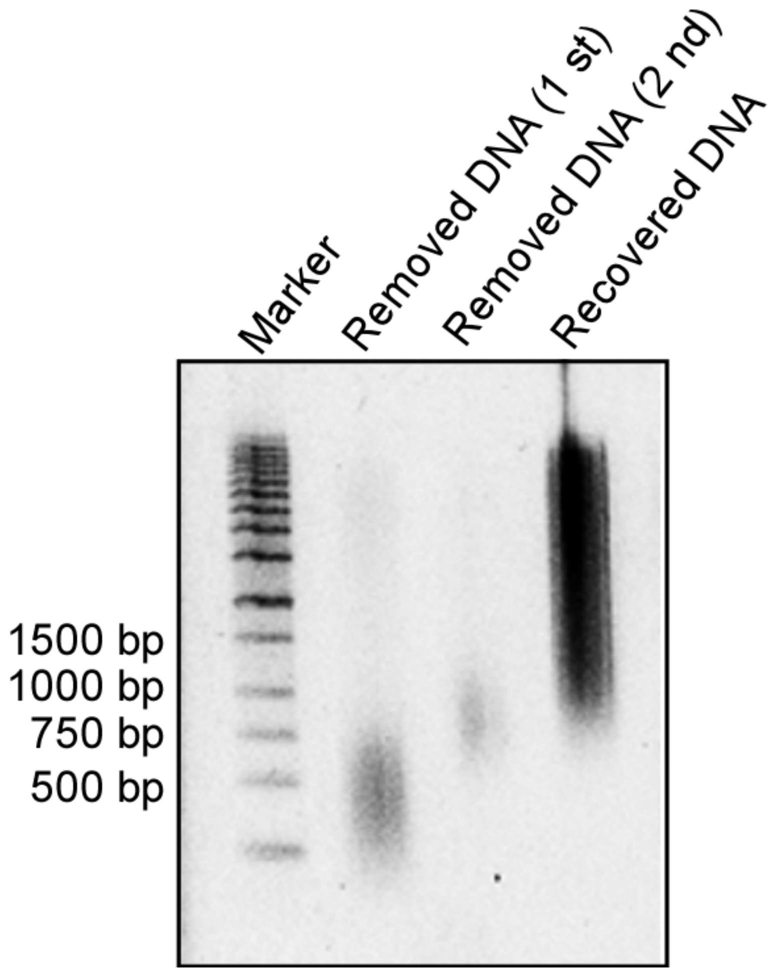

FIGURE S3. Recovery of long-chain DNAs (> 800 bp).
Removed DNAs and recovered DNAs were electrophoresed on 0.7% (w/v) agarose gel. The gel was stained with SYBR Gold (Thermo Fisher Scientific) and analyzed with Gel Doc™ EZ System (Bio-Rad).

TABLE S1 Percentage of viable *E. coli* cell after sorting with MoFlo XDP cell sorter. *E. coli* MegaX DH10B T1 Electrocomp Cells were incubated to an OD_{600} of 0.6 in LB medium at 37°C under aerobic conditions. The culture was diluted with PBS buffer (pH 7.2) to an OD_{600} of 0.01. *E. coli* cells (96 cells) were sorted as a single cell into a 96-well plate (163320, Thermo Fisher Scientific) containing 80 μL LB medium. The nozzle tip was 70 μm in diameter and the voltages used are as described. IsoFlow (Beckman Coulter) was used as a sheath solution. The sorted cell was incubated overnight at 37°C.

Voltage (V)	1000	3500
Percentage of viable cell (%)	43	56
